# Self-Drugging by the Public

**Published:** 1901-10-05

**Authors:** 


### The Hospital

A Journal of

The Medical Sciences and Hospital Administration.

Vol. XXXI.—No. 784.] Edited by Sir Henry Burdett, K.C.B., and Solomon C. Smith, M.D., M.R.C.P. [October 5, 1901.

### Self-drugging by the Public.

It has often been pointed out that the introductory addresses delivered at the various schools of medicine, even if they are not likely to be of any great or abiding value to the students, yet nevertheless, and perhaps even the more completely, afford valuable opportunities of speaking to the public upon questions which greatly concern them. The audiences are to some extent composed of non-medical people, such as parents or guardians who have been desirous to see for themselves the first step in the new career upon which their children or wards were about to enter; and the addresses are liberally reported, and often made the subjects of more or less enlightened comment, in the columns of the daily and weekly non-medical press. The medical profession, engrossed by the immediate duties of the day, or occupied, to quote the language of Sir James Paget, in devising sham remedies for the imaginary grievances of some of its members, has totally neglected the instruction of its employers, the public, and has left the great majority of the classes who would be described as "educated" in the undisturbed enjoyment of the beliefs and notions, with regard to diseases and to "remedies," which they received in childhood from their nursery-maids, who, in their turn, had received them from their grandmothers. It may even be feared that the profession has itself contributed to the continued maintenance of such a state of mind by the careless and frequent use of words and phrases, such as "constitution," and the like, in which the aforesaid grandmotherly and nursery-maid conceptions have become crystallised; with the not unnatural result that it is as impossible, in a large number of instances, to render a pathological doctrine intelligible to the public as it would be to explain the binomial theorem to a savage whose powers of calculation were limited to counting ten upon his fingers. It may even be questioned whether medical men themselves, as a rule, have any adequate conception of the abysmal profundity of the ignorance about all medical questions of the majority of those to whom they are called upon to speak concerning them, or of the extent to which a word, to which absolutely no meaning is attached by most of those who hear it, may baffle and mislead many to whom it is intended to convey instruction. A gallant officer, for example, who has lately been airing in the *Times* paper, at great length and with much repetition, his incapacity to understand the most simple facts bearing upon the possibilities of recovery from pulmonary tuberculosis, would probably find no difficulty in sharing the belief of the Devonshire peasantry, that to carry a potato in the pocket was a sovereign preventive against "rheumatism;" just as he would no doubt regard "rheumatism," in the language of Miss Nightingale, as an entity, like a dog or a cat, which could be picked up and brought to the light for examination and recognition, or which, like either of those animals, could be fostered by appropriate food and other conditions of living, or poisoned, within the body of its proprietor, by something which he would describe as a "remedy" or a "cure."

Dr. Luff, who has this year delivered the introductory address to the students of the Pharmaceutical Society, has availed himself of the opportunity to say a few words of much-needed warning with regard to one of the evil habits which have been fostered by the ignorance to which we have referred—the habit, namely, of taking powerful drugs in unknown quantities without medical advice, and equally without necessity, or even occasion. The blatant advertiser has succeeded in convincing large numbers of people that they may swallow quantities of various condensed or compressed medicines not only without injury, but even with a reasonable prospect of benefit; and Dr. Luff attributes the prevalence of "the comparatively modern and excessively pernicious evil, the cocaine habit," to the ease with which powerful drugs put into these forms can be obtained by the public. He speaks with equal plainness concerning the various proprietary preparations, with the samples and laudatory advertisements of which members of the medical profession are profusely deluged, and says that many of them are, he believes, productive of infinitely more harm than the quack medicines which to some extent they are replacing, for the former are frequently powerful, and, in unskilled hands, dangerous drugs, while the latter, though generally worthless, are to a great extent innocuous. He dwells with much force upon that "absurd fetish," the uric acid diathesis, and the consequent pandering to this modern craze by the unscrupulous vaunters of the many puffed remedies which are warranted to sweep away what is but a natural constituent of the human body. That absurd craze, he tells us, is fostered, if not frequently originated, by the specious advertisements of drugs warranted to cure ills ignorantly, if not falsely, attributable to uric acid, until it has become no uncommon thing at a dinner party to see neurotic young men dropping their lithia tablet into a glass of champagne to counteract what they imagine to be its acidity ; lacking as they do both the gastric vigour to deal with the wine and the moral vigour to abstain from it. It would be a matter of wholesome instruction to many of these drug consumers if they could be made aware of the general fear of taking medicine which prevails among the members of the medical profession itself, to whom, in the majority of instances, a "dose" of anything not absolutely inert is not swallowed without many searchings of heart. We have heard of a gallant officer who told his physician that he had been taking a quack remedy, and asked what he thought of him? To this the physician replied, "I should have thought you entitled to the Victoria Cross."

